# An ENU-Mutagenesis Screen in the Mouse: Identification of Novel Developmental Gene Functions

**DOI:** 10.1371/journal.pone.0019357

**Published:** 2011-04-29

**Authors:** Carolien Wansleeben, Léon van Gurp, Harma Feitsma, Carla Kroon, Ester Rieter, Marlies Verberne, Victor Guryev, Edwin Cuppen, Frits Meijlink

**Affiliations:** Hubrecht Institute, Royal Netherlands Academy of Arts and Sciences and University Medical Centre Utrecht, Utrecht, The Netherlands; Temasek Life Sciences Laboratory, Singapore

## Abstract

**Background:**

Mutagenesis screens in the mouse have been proven useful for the identification of novel gene functions and generation of interesting mutant alleles. Here we describe a phenotype-based screen for recessive mutations affecting embryonic development.

**Methodology/Principal Findings:**

Mice were mutagenized with N-ethyl-N-nitrosurea (ENU) and following incrossing the offspring, embryos were analyzed at embryonic day 10.5. Mutant phenotypes that arose in our screen include cardiac and nuchal edema, neural tube defects, situs inversus of the heart, posterior truncation and the absence of limbs and lungs. We isolated amongst others novel mutant alleles for *Dll1*, *Ptprb*, *Plexin*-*B2, Fgf10, Wnt3a, Ncx1, Scrib*(*Scrib, Scribbled* homolog [*Drosophila*]) and *Sec24b*. We found both nonsense alleles leading to severe protein truncations and mutants with single-amino acid substitutions that are informative at a molecular level. Novel findings include an ectopic neural tube in our Dll1 mutant and lung defects in the planar cell polarity mutants for *Sec24b* and *Scrib*.

**Conclusions/Significance:**

Using a forward genetics approach, we have generated a number of novel mutant alleles that are linked to disturbed morphogenesis during development.

## Introduction

In the developing embryo common molecular pathways are used to generate different cell types, tissues and organs. Although many of these pathways are reasonably well understood, not all of their components are known. Genetic approaches have been of decisive importance in discovery of these factors. Originally, genetic analysis depended on mutations occurring accidentally, but during the last two decades researchers have become less dependent on chance as reverse genetic approaches using targeted gene knock-out strategies have been used to study gene function in the mouse [1]. At the same time, the efficiency of forward mutagenesis screens has increased thanks to technical development, making it applicable not only to micro-organisms and invertebrates but also to more complex organisms.

While in principle reverse genetics makes possible any imaginable mutation, it is laborious, based on assumptions on a gene's function, and may result in an organism with no detectably affected phenotype. On the other hand, a forward genetics approach is unbiased, technically relatively simple and avoids the risk of an unproductive lack of interesting phenotype by selecting affected phenotypes. Moreover, the commonly used chemical mutagen, ENU induces point mutations, frequently leading to hypomorphic phenotypes. Hypomorphic mutations are often informative even for functions of genes of which previously null mutants have been described. Moreover, these point mutations often result in only slightly modified proteins, making the mutant studies informative at a molecular/biochemical level. Advances in sequencing technology are among the most important factors that have contributed to the feasibility of forward screens in complex organisms. The introduction of high-throughput sequencing techniques and the development of databases containing genomic sequences and single nucleotide polymorphism (SNP) of several inbred mouse strains have allowed the generation of SNP panels that facilitate efficient mapping of mutants and subsequent gene identification by sequence analysis.

In a number of different labs mouse forward screens have been conducted and shown to be a fruitful method for the identification of novel gene functions even in mouse [2], [3], [4], [5], [6]. After administration of the mutagen N-Ethyl-N-nitrosourea (ENU) high mutation rates occur in the pre-meiotic spermatogonial stem cells, [7]. ENU acts by transferring its ethyl group onto the bases, transitions at A-T base pairs being the most commonly found mutations [4], [8]. In many cases these screens were performed genome-wide, whereas in other cases they were aimed at a specific genomic region e.g, [9]. An unavoidable aspect of screens in the mouse, it being a viviparous organism, is the choice one has to make of the embryonic stage of analysis. The seminal screen of Kasarskis et al. [10] focused on embryonic day 9.5 embryos, while in the screen by Herron et al. [11] fetuses of E18.5 day were analyzed. While early-lethal mutations do not show up in the latter screen, in the former no mutants with phenotypes that become evident at later stages are detected. A different approach to further focusing on specific potential phenotypes was applied by Zarbalis et al. [5], who used disturbed expression of a Dlx5/6-LacZ transgene as screening criterion.

In the present paper we describe a genome-wide screen aimed at the identification of mutations affecting mouse development, in which we have chosen stage E10.5 for analysis. Although this is only one day later than in the screen of Kasarskis et al.[10], we identified a comparatively quite different set of mutants.

We identified novel gene functions and isolated a series of novel mutant alleles for genes that were previously linked to mutant phenotypes. Three mutants isolated in this screen have been published elsewhere [12], [13].

## Results

### Identified mutants

**Figure 1:**
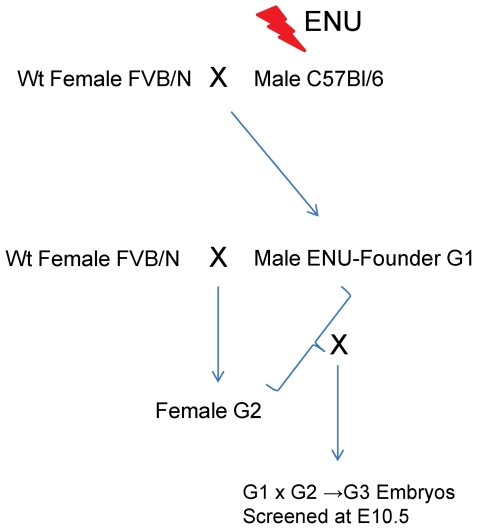
Schematic diagram depicting the ENU-mutagenesis screen. Male C57BL/6 inbred mice were treated with ENU and crossed to FVB/N females to generate 150 G1 founder males. These were subsequently crossed with FVB/N females and the resulting G2 females were backcrossed with their G1 father. The offspring (G3) was screened for disturbed phenotypes at E10.5.

**Figure 2:**
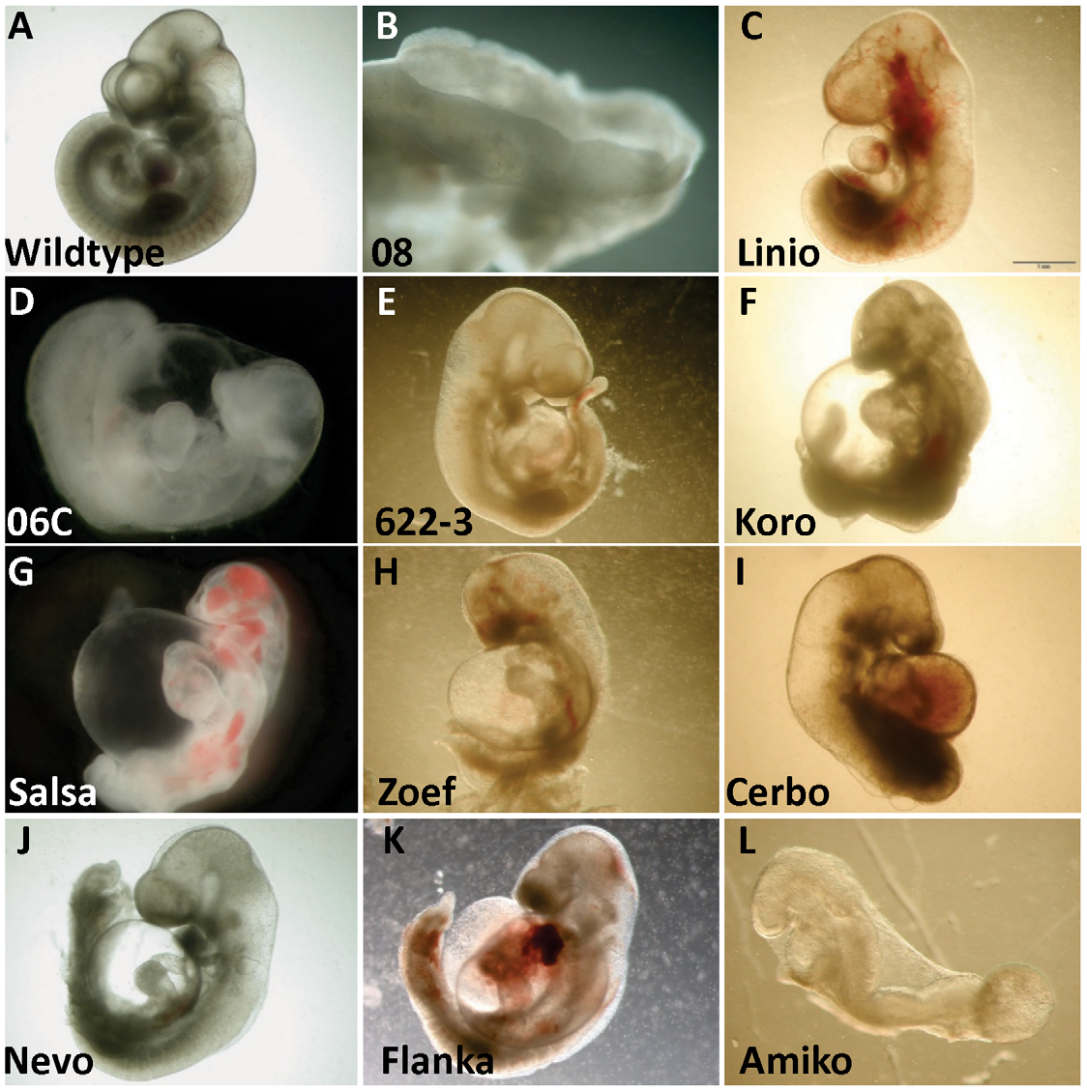
Mutants identified in a recessive ENU-mutagenesis screen. A wildtype (A) embryo and the phenotypes of mutants indentified in the screen for which we thus far have not identified the causative mutation (B-L).

We analyzed the offspring of 150 G1 founders at embryonic day (E) 10.5 (Figure 1), a crucial stage for the development of the heart, limbs and neural tube. We identified at least 25 mutant lines characterized by a variety of reproducible phenotypes, including cardiac and nuchal edema, neural tube defects, a truncated anterior-posterior axis, situs inversus or the absence of limbs and lungs (Table 1; Figure 2). An exact number of mutant lines obtained is hard to give, as we terminated analysis of a number of potentially genuine mutant lines when mapping data were too confusing or reproducibility of the phenotype was doubtful. Therefore we can only give a minimum estimate. Amongst the mutants described included in Table 1 are several for which we have not yet identified the causative mutation.

**Table 1:** Overview of mutants found in this screen (see Table S2 for additional information).

Line^a^	Phenotype^b^	Chr	Mbp^c^	Affected gene	Mutation	Figure	Ref.
5120-6B	Craniorachischisis	15	71-83.2	Scribble			[12]
5120-6C	Cardiac edema	10	25-29	Unknown		2D	
5120-7	Cardiac edema	17	78–84.5	Ncx1	N874K		[13]
5120-8	Open hindbrain	11	115–120	Unknown		2B	
59458-3	Craniorachischisis	3	121.6–130.8	Sec24b			[12]
59459-2	Situs inversus and short tail	17	5.5–27.5	Dll1	E26G	3	
59468-4	Cardiac edema	10	114–116.5	Ptprb	Y693X	4	
59622-3	Cardiac edema	3	49–76	Unknown		2E	
59780-4	NTD fore- and midbrain	15	8–89.4	PlexinB2	E369G	5	
Amiko	Growth arrest at E9.0	14	24–72	Unknown		2L	
Cerbo	Cardiac and nuchal edema	2	165–166	Unknown		2I	
Flanka	Abnormal head, heart, NTD	6	14.1–32.2	Unknown		2K	
Koro	Cardiac edema	11	3.2–17.6	Unknown		2F	
Pootloos	No limbs	13	-	Fgf10	L91P	6	
Linio	Cardiac edema	11	94–98.7	Unknown		2C	
Nevo	Cardiac and nuchal edema	8	77.4–98	Unknown		2J	
Salsa	Cardiac edema	6	67–71	Unknown		2G	
Staartloos	Posterior truncation	11	55–66	Wnt3a	unknown	7	
Zoef	Cardiac and nuchal edema	19	33.5–33.8	Unknown		2H	

a Trivial name of line.

b Major distinctive phenotype.

c Candidate interval after mapping.

Optimal ENU induction in mice is expected to lead to a mutation rate of approximately one mutation per 1.5 mega base pairs (Mbp) [14]. Based on this mutation rate, a size of the mouse genome of approximately 2.5×10^3^ Mbp and assuming that approximately 1.5% of the genome encodes a protein, we anticipate that each G1 founder contains about 25 mutations that affect protein sequence.

### A substitution mutation in *Dll1* leads to disturbed left-right patterning and axial truncation in *59459-2* mutants

**Figure 3:**
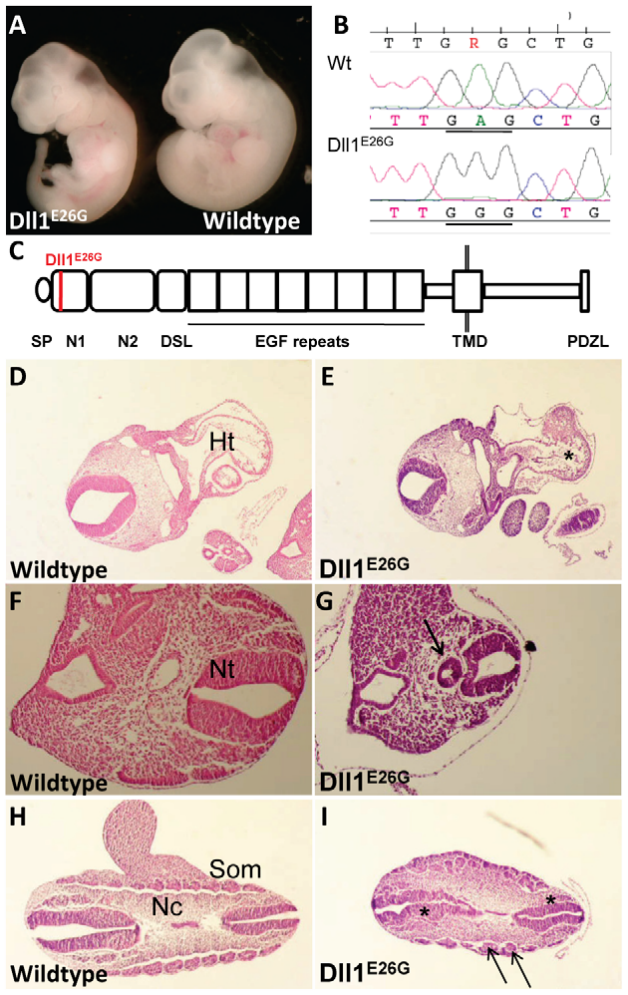
A substitution mutation in *Dll1* leads to situs inversus and posterior truncation. (A) Whole mount view of *Dll1^E26G^* and wildtype embryos. Note the shorter tail in the mutant. (B) An A-to-G mutation causing a glutamine-to-glycine substitution in the Dll1^E26G^ mutant protein. (C) The mutation is localized in the N1 domain. SP, signal peptide; N1, N-terminal domain 1; N2, N-terminal domain 2; DSL, Delta-Serrate-Lag2 domain; EGF repeat, epidermal growth factor-like repeats; TMD, transmembrane domain; PDZL, PDZ (postsynaptic density 95, PSD-85; discs large, Dlg; zonula occludens-1, ZO-1) ligand motif. (D-I) H&E stainings on transverse sections at E9.5. The asterisk in E marks reversed looping compared to wildtype (D). NT, neural tube; Ht, heart. The arrow in G indicates an ectopic neural tube (F). Irregularly shaped neural tube (asterisks) and somites (arrows) in Dll1E26G mutants (H,I). Som, somites; Nc, notochord.

A mutant characterized by a short tail as well as heart defects including situs inversus (Figure 3A), was mapped to Chromosome (Chr) 17 (5.5–27.5 Mbp). Exons of genes in this interval were sequenced, which resulted in the discovery of an A to G transition (Figure 3B) in the gene encoding the Notch DSL ligand Delta-like1 (*Dll1*). This mutation, Dll1^E26G^, causes a glutamine-to-glycine substitution at amino acid position 26 (ENSMUSG00000014773). Glutamine 26 is highly conserved between species as well as in the Dll1-related protein Jagged/Serrate [15] and is positioned in the N-terminal domain 1 of the protein (Figure 3C).

*Dll1* is known to be required for the formation and identity maintenance of the caudal somites [16] and for left-right asymmetry in the embryo [17]. *Dll1* expression starts during the mid-streak stage in embryonic mesoderm. In the late-streak embryo expression is restricted to the posterior mesoderm, but it is absent from the node. At the 5-somite stage it is expressed in the presomitic mesoderm, the caudal halves of the condensed somites and in the neuroepithelium of the presumptive midbrain region [18].

*Dll1^E26G^* mutant embryos were posteriorly truncated (Figure 3A). Similar shortened anteroposterior axes have been previously reported for *Dll1* mutants by Hrabé de Angelis et al. [19]. In addition the neural tube and the somites were irregularly shaped in the posterior part of mutant embryos (Figure 3H,I). Interestingly, an ectopic neural tube was occasionally present in *Dll1^E26G^*mutant embryos, ventrally of the primary neural tube and dorsally of the notochord (Figure 3F,G). *Tbx6* mutant embryos often display an ectopic neural tube, which is accompanied by decreased levels of *Dll1* expression in the mesoderm [20]. We are however not aware of similar reports for the *Dll1* mutant itself. Finally, we observed randomized heart looping in *Dll1^E26G^* mutant embryos (Figure 3D,E) as previously noted by Przemeck et al. [17] for the previously characterized *Dll1* mutant.

Notch signaling has been implicated in the development of the left-right axis, and heart looping and turning of the embryoare randomized in *Dll1* mutants. This phenotype originates from the node, which displays morphological abnormalities in these mutants, including loss of monociliated cells [17]. The strong resemblance to the known knock-out null phenotype that the *Dll1^E26G^* embryos display indicates that the amino acid substitution observed is sufficient to abolish most or all of the biological function of the protein. A likely explanation is that Dll1E26 is essential in the binding of Dll1 to the Notch receptors, since the two N-terminal domains together with the DSL domain are responsible for the binding of DSL ligands to the Notch receptors [15], [21], [22]. The combination of mapping data with the similar phenotypes between *Dll1^E26G^* mutant embryos and the previously described *Dll1* mutant makes allelism between *59459-2* and *Dll1* very likely.

### A premature stop codon in *Ptprb/VE-PTP* leads to early embryonic lethality in *59468-4* mutants

**Figure 4:**
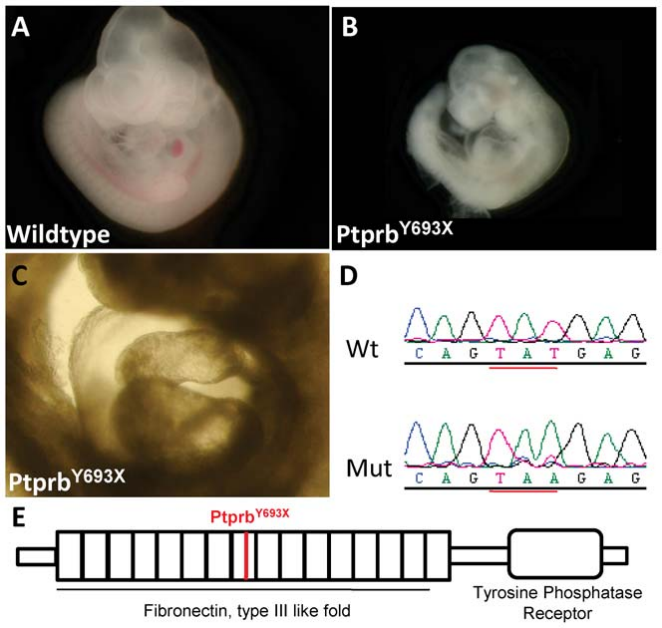
Cardiac edema in a mutant for *Ptprb.* Comparison of wildtype (A) and Ptprb mutant embryos (B,C). Note the cardiac edema in C. Sequence analysis shows a T to A mutation in Ptprb (D). Location of the Ptprb^Y693X^ mutation (E).

The *59468-4* mutant exhibits slight cardiac edema (Figure 4a–c). Genome-wide mapping linked it to a region on Chr 10 between 114 and 116.5 Mbp. We noticed a striking resemblance to the phenotype reported for mutants of one of the genes present in this interval, Ptprb, also known as E*-PTP*. *Ptprb/VE-PTP* mutants have previously been described as being defective in angiogenesis; vascular remodeling defects lead to a severely inflated pericardial sac and growth arrest at embryonic day 9.5 [23], [24]. Baumer et al. also report that in their mutants the endocardium fails to attach to the myocardium, eventually leading to trabeculation defects; furthermore, they observed failure of intersomitic vessel development. These malformations strongly correlate with the expression pattern of *Ptprb/VE-PTP* mRNA, which is throughout development predominantly localized to atrial endothelium [24]. VE-PTP acts by negatively regulating Tie2, a protein that regulates endothelial cell proliferation and thus blood vessel remodeling [25].

The *Ptprb* encoding tyrosine phosphatase receptor b is located within the region on Chr 10 mentioned above, and sequencing of its coding regions revealed a T to A transversion at nucleotide position 2079 (ENSMUST00000020363) (Figure 4D). This mutation causes a stop codon at the tyrosine at position 445 of this 1998-amino acid long protein (Figure 4E). This early stop codon ablates the protein tyrosine phosphatase domain in the C-terminal part of the protein and likely leads to total loss of protein function and therefore a null mutant. Allelism between *59468-4* and *Ptprb/VE-PTP* is not formally proven by these results, but is highly likely in view of the combination of independent types of evidence, i.e. similarity of phenotypes and mapping.

### Exencephaly in the *59780-4* mutant is caused by a glutamic acid to glycine substitution in the Sema-domain of Plexin-B2

**Figure 5:**
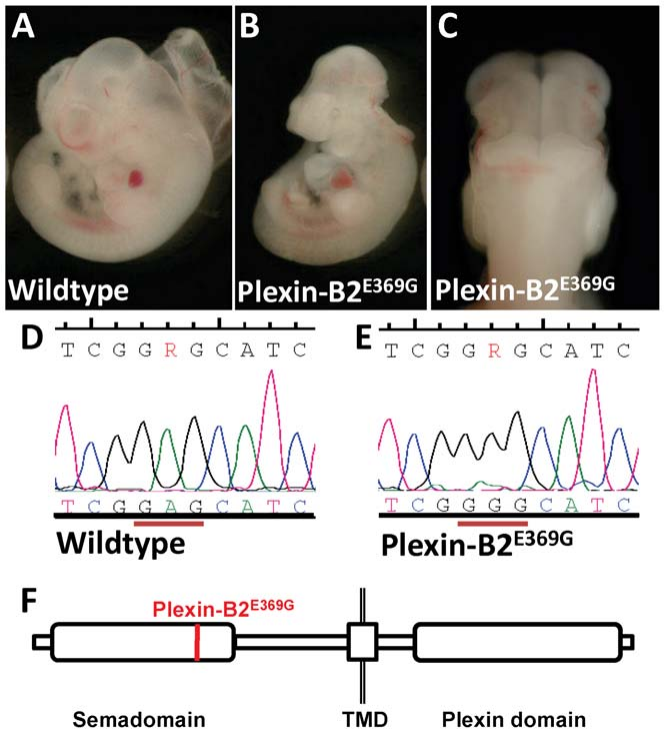
A mutation in *Plexin-B2* leads to exencephaly. Mutant embryos displaying closure defects throughout the brain region of the neural tube (A–C). Sequencing revealed an A-to-G point-mutation (D). Location of the Plexin-B2^E396G^ mutation (E).

A mutant displaying exencephaly (Figure 5A–C) was mapped to chromosome 15 between 88.0 and 89.4 Mbp. Exons from the genes located in this segment were sequenced and the *Plexin-B2* gene was found to contain an A to G transition only in DNA from mutants. This mutation predicts a glutamic acid to glycine substitution at amino acid position 369 E369G (Figure 5D,E). The membrane receptor Plexin-B2 is expressed in proliferating granule cell progenitors. Plexins are the receptors for Semaphorins; these ligands are involved in processes underlying proliferation, differentiation and migration in a variety of tissues [26].

*Plexin-B2* mutants have been reported previously; they die at birth due to neural tube closure defects extending from fore- to hindbrain. [27], [28]. No molecular mechanism explaining this phenotype has been put forward. In the few mutants of the KO line that do not show a neural tube closure defect and survive until after birth, *Plexin-B2* has been reported to have a role in maintaining the balance between differentiation and proliferation of the granule cells. Loss of this balance in *Plexin-B2* mutants was said to result in over-proliferation of the differentiated granule cells that migrate into the cerebellum, leading to a severely altered cerebellar cytoarchitecture [27], [28].

The amino acid that is changed in our *Plexin-B2^E369^*mutant is localized in the Semaphorin-binding domain of the protein (Fig 5F), which may indicate that the glutamic acid at this position is essential for the binding of these Semaphorins to the cell surface receptor Plexin-B2. This possibility has not yet been tested. Given the combination of independent evidence, allelism of *59780-4* and *Plexin-B2* is very likely, but remains formally unproven.

### A leucine to proline substitution in FGF10 leads to absence of limbs and lungs in *Pootloos* mutants

**Figure 6:**
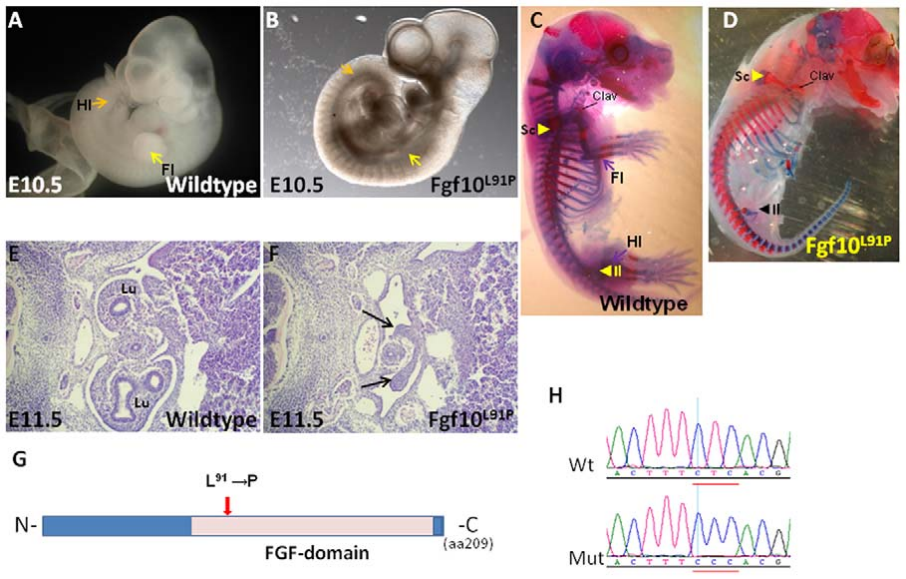
A leucine-to-proline substitution in the FGF domain of FGF10 leads to absence of limbs and lungs. Wildtype E10.5 embryo (A) and mutant embryo (B) displaying lack of limb buds at E10.5. In (A), yellow and orange arrows indicate location of fore and hind limbs (FL, HL), respectively, and similar arrows indicate in (B) the areas where they would have been expected to appear. (C,D) Comparison by bone stainingof wildtype (C) and mutant (D) FGF^L91P^ E15.5 embryos. In the mutant, clavicle (Clav), parts of the scapulae (Sc) and rudimentary iliac bones (Il) are present, but fore- and hind limb bones are absent. Sequencing revealed a T-to-C mutation (D). (E,F) Histological sections of E11.5 wildtype (E) and mutant (F) embryos at a thoracic level. ‘Lu’ in (F) indicates the lungs and arrows in G indicate the undeveloped lung buds in the mutant embryo. (G) Schematic representation of the alteration in the FGF protein, based on the detected mutation (H).

The *Pootloos* mutant (Figure 6) was found on the basis of it lacking limb buds at E10.5 (Figure 6B,D). Alcian Blue and Alizarin Red bone stainings at a later stage (Figure 6C,D) show the presence of the clavicle and anterior scapula. In the pelvic region, a rudimentary iliac bone was present but the fore- and hind limb bones are absent (Figure 6C,D). Transverse sections of E11.5 mutant embryos revealed the presence of primary lung buds, but no outgrowth and branching of the lungs (Figure 6E,F). This phenotype strikingly resembles the unique total loss-of-function phenotype of *Fgf10* mutants described by Min et al. and Sekine et al. [29], [30]. The mutants described by these authors are also characterized by the absence of limbs, retaining clavicle, anterior scapula and a rudimentary iliac bone. They have been reported to die shortly after birth due to the absence of the lungs [29], [30]. After confirming linkage of the mutation to *Fgf10* by limited mapping using a small number of SNPs, we proceeded by sequencing the Fgf10-coding region of *Pootloos* mutants and wildtypes. By exception, in this case the other candidates in this region were not sequenced. We identified a T to C transition predicted to cause a leucine to proline substitution at amino acid position 91 (ENSMUSP00000020363) (Figure 6H). This amino acid is part of a conserved domain common to all FGFs, but does not appear to be involved directly with functions characteristic of FGFs, like interactions with the FGF receptor or with heparin (see: http://www.ncbi.nlm.nih.gov/Structure/index.shtml; [31]; Figure 6G). The insertion of a proline is likely to have a major impact on essential aspects of the protein structure of the FGF-domain and may cause a total loss-of-function. The combination of independent evidence from a unique phenotype, the partial mapping and the presence of the mutation makes allelism of *Fgf10* and *Pootloos* extremely likely.

### Severe axial truncation in the *Staartloos* mutant

**Figure 7:**
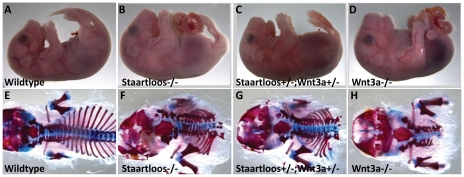
Genetic interaction of *Staartloos* with *Wnt3a*. (A–D) Whole mount views of wildtype (A), *Staartloos* homozygous (B), *Staartloos/Wnt3a* transheterozygous (C) and *Wnt3a* KO mutant embryos (D). (E–H) Bone stainings of wildtype (E), *Staartloos* homozygous (F), *Staartloos/Wnt3a* double heterozygous (G) and *Wnt3a* KO mutants (H).

One mutant, named *Staartloos,* displaying a severe posterior truncation defect was found in the screen. Homozygous embryos had no tail, sometimes lacked hind limbs or had fused hind limbs (sirenomelia), and lacked caudal somites or vertebrae (Figure 7A,B). Mapping resulted in a candidate interval on chromosome 11 between 55 and 66 Mbp. All genes in this area were sequenced, but no mutations were found. The phenotype of this mutant line shows striking resemblances to the previously characterized knock-out phenotype of one of the genes present in the candidate interval. *Wnt3a* mutants have also been described as posteriorly truncated, a complex of defects including disrupted development of notochord and tailbud and lack of caudal somites [32], [33], based on failure of axial extension during early development. A striking phenotype previously reported for other *Wnt3a* mutant alleles is the occurrence of a partially duplicated neural tube. In work with *Staartloos* embryos, this phenotype has recently also been demonstrated (J. Deschamps, personal communication). Sequencing of a Wnt3a cDNA clone from a homozygous mutant, the coding region of genomic DNA, exon/intron transitions, 5′- untranslated regions neither revealed a mutation. We therefore crossed the *Staartloos* mutant with the *Wnt3a* KO mice [32] to explore non-complementarity of these two mutations. Double heterozygous mutant embryos were very similar to *Staartloos* mutants (Figure 7B,C), confirming that *Staartloos* carries a *Wnt3a* mutant allele. Possibly *Wnt3a* levels in the mutant are affected by a mutation in a regulatory element. Intriguingly, the causative mutation for the *Wnt3a* hypomorphic mutant *Vestigial tail* has never been reported either. In embryos homozygous for the *Vestigial tail* mutation,*Wnt3a* levels are reduced, suggesting that the mutation affects regulation of *Wnt3a*. *Staartloos* mutants contain more caudal vertebrae than Wnt3A KO embryos (Figure 7E-H), suggesting that the *Staartloos* mutation represents a hypomorphic condition for *Wnt3a*. Allelism of *Staartloos* with *Wnt3a* is therefore supported by four types of evidence: (i) genome-wide mapping; (ii) a phenotype very similar to that described in two mutant alleles of *Wnt3a*; (iii) a failure of the mutant to complement the *Wnt3a* phenotype. Therefore *Staartloos* is almost certainly allelic with *Wnt3a.*

### Seven mutant lines carrying the same mutation

Seven mutants linked to an identical cardiac edema phenotype mapped in the same region of Chromosome 6 between 67 and 71 Mbp. Compound mutants of several combinations of these lines all led to the same phenotype, demonstrating that the same locus was affected. These lines must all have derived from a single spontaneous mutation present in some of the C57BL/6 mice used for the ENU injections. We have combined these mutants under the name *Salsa* (Table 1 and Fig. 2) and are still in the progress of identifying the causative mutation.

### Affected lung development in two PCP mutants

**Figure 8:**
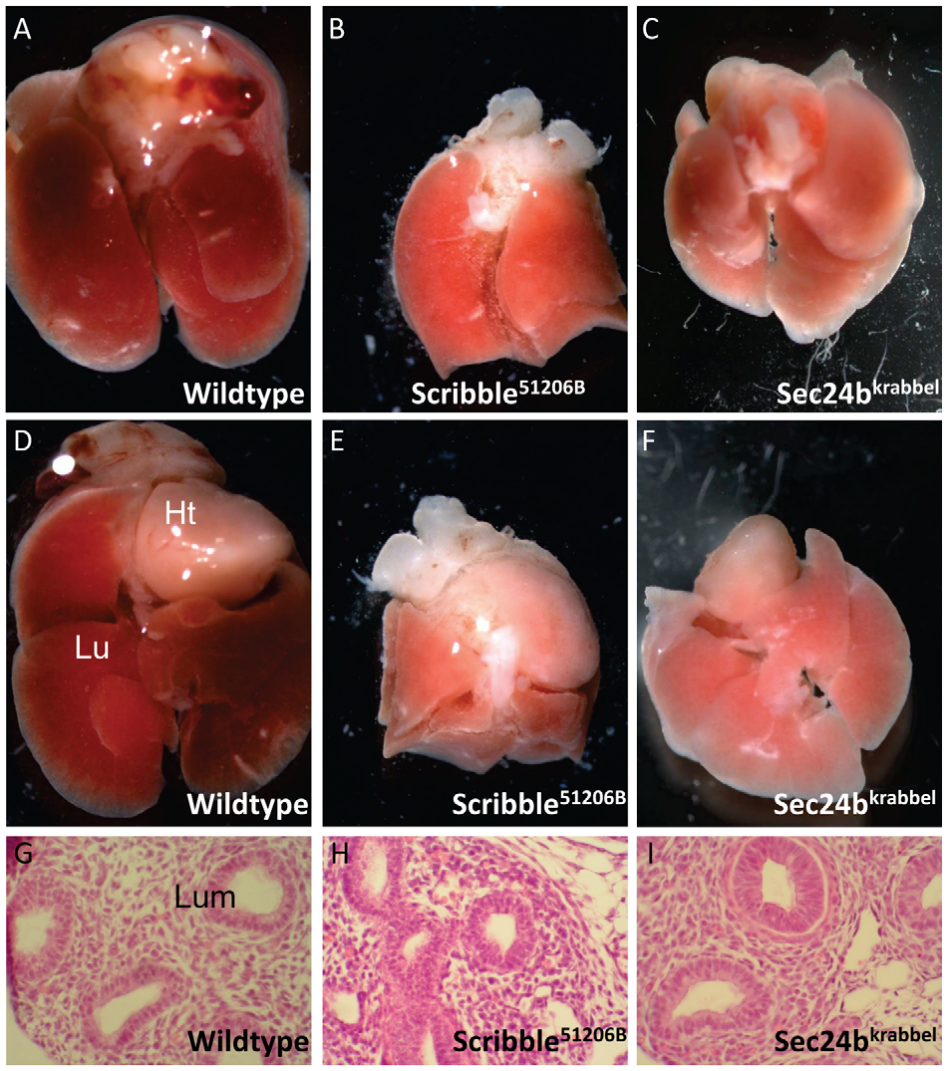
Affected lung development in the two PCP mutants obtained in our screen. (A–C) Dorsal and (D–E) ventral views of the lungs and hearts of (A,D) wildtype (B,E) *Scrib* and (C,F) *Sec24b* mutant embryos. Note the reduction in size of the mutant lung, in particular that of *Scrib*. (G–I) Sections of wildtype (G), *Scrib* mutant (H) and *Sec24b* mutant (I) lungs at E13.5. Ht and Lu (D) mark the heart and Lungs respectively, lumina (G) are labeled ‘Lum’.

We identified two mutants in our screen that were characterized by the neural tube defect craniorachischisis in homozygous mutant embryos, *Sec24b^Krabbel^* and *Scrib^5120-6B^*; see [12]. In addition to the defects described above, we observed a lung development defect. At E17.5, the lungs of both mutants are smaller than those of wildtypes and the lobes are irregularly shaped (Figure 8A–F), with a clearly more severe phenotype seen in the *Scrib* mutant (Figure 8B,E; compare to Figure 8C,F). As we have shown that Sec24b is essential for adequate intracellular trafficking of Vangl2 in the neural tube [12], it is tempting to speculate that the lung phenotype in the *Sec24b* mutant is also caused by lower levels of Vangl2 at the cell membrane.

## Discussion

In this phenotype-based screen we have identified a series of new mutant alleles causing disrupted embryonic development. In some cases these new mutants represent unexpected genotype-phenotype associations while in other cases comparatively subtle mutations in previously studied genes reveal new findings at a molecular level.

The efficiency of our screen has been of similar efficiency compared to the comparable screens in the Anderson group (e.g., [3] [10]). Kasarskis et al. [10] mention that 5–10% of founders tested showed a reproducible affected phenotype, and our data are in the same range. Exact meaningful numbers are difficult to give, since about 35 of the 150 founder lines were abandoned when we deemed the phenotype insufficiently reproducible, or when the initial mapping yielded unclear data; some of these lines may have represented real mutants. Of about 115 founders at least 4 G3 litters containing at least 30 embryos were analyzed.

We have no explanation for the fact that so few mutants identified by us display a defect linked to Sonic hedgehog signaling, as was the case in the screens mentioned above. Many of our mutants were identified by cardiac edema. This perhaps reflects the stage that we chose for the initial analysis of potentially mutant embryos, which is 1–2 days after the embryo becomes dependent on a functioning beating heart. In addition, cardiac edema is an endpoint of many different lethal defects.

Unexpected findings included the identification of a *Scrib* allele that must have arisen due to a ‘spontaneous’ (i.e. not ENU-induced) mutation of an FVB/N female, and the confusing findings with the *Salsa* mutant. In this case mutations were found that initially appeared to explain the phenotype; however the mutations were also present in some of the heterozygotes and must have been derived from genetic variation in the CB57/B6 mice used. This underscores the importance of checking the genetic purity of the mice strain used for the mutagenesis.

### PCP signaling and lung development

Scribble, Vangl2 and Sec24b have all been linked to the planar cell polarity (PCP) pathway that regulates the polarity in the plane of a sheet of epithelial cells and controls convergent extension of a tissue [12]; reviewed by [34]. The involvement of PCP signaling in lung development is supported by the following data: (i) In vitro lung branching is under the control of the downstream PCP factor RhoA [35]. (ii) Lung defects have been reported in mouse mutants for the ‘non-canonical’ *Wnt5a* gene [36]. Furthermore, over- and ectopic expression of Wnt5a in a cultured chick lung explant model confirmed its importance for lung development [37] and (iii) mutants for the PCP-related gen *Fat4* [38] display branching defects in the kidney, an organ that shares similar signaling pathways with the lung and other organs containing branching tubes. These observations make it worthwhile further investigating the possible role of noncanonical Wnt signaling in lung development (see also: [39]). Recently Yates et al. [40] for the first time linked the core PCP pathway to lung development; they showed an abnormal size and shape of the lung lobes in *Celsr1* and *Vangl2* mutants. Both mutants have defects in lung branching and abnormalities of cytoskeletal architecture, as well as a reduced number and width of the airway lumina. The phenotype was confirmed in a lung explant model and a reduced response to FGF10 was demonstrated in this context. In vitro activation of Rho kinase restores lung branching in the *Vangl2* and *Celsr1* mutants. In addition to this, *Celsr1* has a specific role in bifurcation of the lung buds. These authors also mention that they found a similar phenotype in their *Scribble* mutant [40]. We found, in addition to the small and irregular lung lobes, a disturbed cellular organization in the lumina in the *Sec24b* and *Scribble* mutants (Figure 3H,I), comparable to the double layers of epithelial cells in the *Celsr1* and *Vangl2* mutants [40]. Possibly, Sec24b and Scribble also direct lung development through the PCP pathway. The strong phenotype in the Scrib^5120*-6B*^ mutant opens the possibility that there might be another crucial role for Scribble in lung development.

### Conclusions

While reverse genetics (i.e., gene targeting technology) is powerful in that theoretically any desired genotype can be created, forward genetics is valuable as a parallel system and has a number of advantages: (i) the unbiased approach to identify genes of unknown function involved in development; (ii) the identification of single amino acids changes that alter protein function and (iii) the identification of novel phenotypes and functions of genes of known function that bypass lethality by the introduction of hypomorphic substitution mutations. A case in point for (i) is the *Sec24b* mutant, where we found the surprising link between Sec24b-dependent COPII-coated endoplasmic reticulum to Golgi protein transport, Vangl2 trafficking and PCP[12]. Examples of (ii) are the various mutants described in this paper in which a single missense mutation leads to a very strong phenotype, demonstrating *in vivo* the biochemical essentiality of the residue in question.

Recently, technological advances including ‘Next Generation Sequencing’ [41] have further reduced the efforts needed to isolate mutations. This will add greatly to the potential of ENU-mutagenesis screens, since the causative mutations can be found more rapidly. It should be possible to pinpoint the elusive mutations in *Staartloos* and *vestigial tail* Wnt3aalleles in a relatively straight-forward manner, although this will also require analysis of reporter construct to test directly the mutations found.

## Materials and Methods

### Ethics statement

Animal experiments were conducted under the approval of the ‘*dierexperimentencommissie’*(animal care committee) of the Royal Netherlands Academy of Arts and Sciences (KNAW), (permit numbers HL03.0204, HL04.0201, HL05.0201 and HL05.0203).

### ENU-mutagenesis

40 C57BL/6 males were injected three times intraperitoneally with 60–80 mg/kg bodyweight ethyl-N-nitrosourea (ENU) with one-week intervals. After a recovery period of 8-10 weeks the mice were crossed with FVB/N females to generate 150 G1 males. These ‘founder’ males were subsequently crossed with FVB/N to generate G2 females, who were crossed with their G1 father to generate G3 embryos. Each of these embryos is expected to be homozygous for 6.25% of the mutations initially induced in the C57BL/6 mice. These G3 litters were collected at E10.5 and screened for any perceptible malformations. DNA of the mutant and their littermates was isolated for mapping. In rather numerous cases where a presumptive mutant was identified initially on the basis of severe cardiac edema at E10.5, we did further isolations at E9.5, which often allowed more relevant observations on the specific nature of the phenotype and also to avoid possible loss of embryos due to resorption. We screened a minimum of four G3 litters (of at least six, and on average more than eight embryos) produced by each G1 male. All mutant lines described in this paper are available from the authors to interested scientists. Lines were maintained by crossing against FVB/N until the mutation had been mapped. The exception was with the *Scrib^5120-6b^* line that arose from a mutation in a FVB/N mouse used for outbreeding [12].

### DNA isolation

DNA was collected from embryos, yolk sacs (<E9.0) or ear clippings. The DNA was isolated by overnight incubation at 55°C while rotating in lysis buffer [TENS: 100 mMTris-HCl pH 8–8.5, 200 mMNaCl, 0.2% sodium dodecyl sulphate and 5 mMEthylenediaminetetraacetic acid (EDTA)] supplemented with 100 µg/ml proteinase K. From ear clippings hair was removed by centrifugation for 15 min at 12,000 x g. DNA was precipitated by adding 1 volume of 100% isopropanol at room temperature and vortexing for 5 min. To dissolve, the pellet was incubated with TE buffer (10 mMTris pH 7.5, 0.1 mM EDTA) overnight at 55°C while rotating. DNA was stored at 4°C.

### Genetic mapping and genotyping

A panel of 192 SNPs discriminating between FVB/NJ and C57BL/6 DNA alleles was designed (see Table S1). These SNPs are spread equally over the genome and were used for initial genome-wide mapping using approximately 8 mutant and 8 wildtype or heterozygous DNAs isolated from each mutant line. Additional mapping using extra SNPs in the identified regions (http://phenome.jax.org/pub-cgi/phenome/mpdcgi?rtn=docs/home) reduced the candidate regions to intervals small enough to start sequencing coding regions (see Table 1). In a limited number of cases we gave priority to genes in this interval that had been previously characterized and shown to have phenotypes resembling our mutant. Mice and embryos were genotyped by PCR and sequencing of the mutation or SNPs in the identified interval. Generally between 70 and 100% of the genes in the candidate interval was sequenced, depending on the likeliness that found mutations corresponded to the phenotype observed. Table S2 summarizes mapping data for mutant lines discussed in this paper.

### Sequence analysis

To amplify SNPs and exons of genes, a so-called touch-down program was used: 1′ at 94°C followed by 12 cycles of: [30″ at 92°C; 30″ starting at 65°C and 0.6°C lower at each cycle; 30″ at 70°C], followed by 20 cycles of: [30″ at 92°C; 30″ at 58°C; 30″ at 72°C] followed by a final step of 3′ at 72°C. GoTaq polymerase (Promega) was used according to the supplier's instructions. Gene-specific primers were designed using software accessible at http://primers.niob.knaw.nl/ and http://limstill.niob.knaw.nl/.

PCR products were diluted and sequenced using BigDye v3.1 terminator (Applied Biosystems). The following protocol was used: 1′ at 95°C, 30 cycles of 10″ at 95°C, 5″ at 50°C, 2′ at 60°C. After the sequence reaction, the samples were purified using Sephadex G-50 Superfine (Sigma) or by ethanol/EDTA/Sodium Acetate precipitation. Sequencing was done at the Hubrecht institute sequencing facility with a 96-capillary 3730XL DNA analyzer (Applied Biosystems), using the standard RapidSeq protocol on 36 cm array. Mutations were identified using software based on Polyphred [42].

### Miscellaneous procedures

Bone stainings were performed exactly as described by Beverdam et al. [43]. Hematoxylin and eosin staining were done according to standard procedures.

## Supporting Information

Table S1 **A 192-genome wide SNP panel.Listing of SNPs used in the genome-wide panel.** First column, arbitrary number for identification; second column, rs numbers allowing retrieval from common databases (e.g. http://www.ncbi.nlm.nih.gov/projects/SNP/snp_blastByOrg.cgi); third column (‘Chr’), chromosome number; fourth and fifth columns provide expected nucleotide for C57Bl/6 and FVB/N, respectively.
(XLS)

Table S2 **Mapping coordinates of mutant lines.** Rs numbers correspond to the SNP database For each mutant the borders of the candidate region is given by the name (rs number) of the SNP (see http://www.ncbi.nlm.nih.gov/projects/SNP/snp_blastByOrg.cgi) as well as, in the last two columns, the chromosomal coordinates.
(DOCX)
