# Supplementary material for: An ENU-Mutagenesis Screen in the Mouse: Identification of Novel Developmental Gene Functions
Source: PLoS One. 2011 Apr 29;6(4):e19357. doi: 10.1371/journal.pone.0019357 (PMC3084836; doi:10.1371/journal.pone.0019357)
Supplement: Table S2 — Mapping coordinates of mutant lines. Rs numbers correspond to the SNP database For each mutant the borders of the candidate region is given by the name (rs number) of the SNP (see http://www.ncbi.nlm.nih.gov/projects/SNP/snp_blastByOrg.cgi) as well as, in the last two columns, the chromosomal coordinates. (DOCX) [file pone.0019357.s002.docx]

Supplementary File II

Mapping coordinates of mutant lines. ‘rs’ numbers correspond to the SNP database (<http://www.ncbi.nlm.nih.gov/projects/SNP/>)

Mutant SNPs between which mutant has been mapped chromosomal segment

| 06B | rs6169611 | rs4230908 | 15:70046283 | 15:82956832 |
| --- | --- | --- | --- | --- |
| 06C | rs13480554 | rs13480567 | 10:25670693 | 10:28885318 |
| 07 | rs13483120 | rs13483144 | 17:79876068 | 17:86285312 |
| 08 | rs3708178 | rs6280170 | 11:114503379 | 11:120340622 |
| 458-3 | rs6177693 | rs29976476 | 3:121257775 | 3:130722680 |
| 459-2 | rs13482849 | rs33603240 | 17:5612314 | 17:27972030 |
| 468-4 | rs8275399 | rs13473163 | 10:114027796 | 10:116500074 |
| 622-3 | rs13477098 | rs13477215 | 3:48412765 | 3:78034264 |
| 780-4 | rs32094491 | rs3692372 | 15:88007996 | 15:89439195 |
| amiko | rs13482097 | rs13482248 | 14:24572106 | 14:72506628 |
| cerbo | rs27345176 | rs27274584 | 2:165271326 | 2:167274828 |
| flanka | rs13478638 | rs13478695 | 6:14310522 | 6:32292927 |
| koro | rs13480835 | rs26852711 | 11:3234216 | 11:17626504 |
| pootloos | x | x | x | x |
| linio | rs13481166 | rs27026226 | 11:94234188 | 11:98715764 |
| nevo | rs33310964 | rs6356426 | 8:77358152 | 8:97973032 |
| salsa | rs6157367 | rs30648489 | 6:67237174 | 6:71307455 |
| staartloos | x | x | x | x |
| zoef | rs36365181 | rs31284274 | 19:33523824 | 19:38009891 |
